# Multisensory System Used for the Analysis of the Water in the Lower Area of River Danube

**DOI:** 10.3390/nano9060891

**Published:** 2019-06-17

**Authors:** Constantin Apetrei, Catalina Iticescu, Lucian Puiu Georgescu

**Affiliations:** Department of Chemistry, Physics and Environment, The European Centre of Excellence for the Environment, Faculty of Sciences and Environment, “Dunarea de Jos” University of Galati, 800008 Galati, Romania; catalina.iticescu@ugal.ro (C.I.); lucian.georgescu@ugal.ro (L.P.G.)

**Keywords:** water, Danube, sensor, nanomaterial, carbon nanotube, carbon nanofiber, graphene, gold nanoparticle, cyclic voltammetry, data analysis

## Abstract

The present paper describes the development of a multisensory system for the analysis of the natural water in the Danube, water collected in the neighboring area of Galati City. The multisensory system consists of a sensor array made up of six screen-printed sensors based on electroactive compounds (Cobalt phthalocyanine, Meldola’s Blue, Prussian Blue) and nanomaterials (Multi-Walled Carbon Nanotubes, Multi-Walled Graphene, Gold Nanoparticles). The measurements with the sensors array were performed by using cyclic voltammetry. The cyclic voltammograms recorded in the Danube natural water show redox processes related to the electrochemical activity of the compounds in the water samples or of the electro-active compounds in the sensors detector element. These processes are strongly influenced by the composition and physico-chemical properties of the water samples, such as the ionic strength or the pH. The multivariate data analysis was performed by using the principal component analysis (PCA) and the discriminant factor analysis (DFA), the water samples being discriminated according to the collection point. In order to confirm the observed classes, the partial least squares discriminant analysis (PLS-DA) method was used. The classification of the samples according to the collection point could be made accurately and with very few errors. The correlations established between the voltammetric data and the results of the physico-chemical analyses by using the PLS1 method were very good, the correlation coefficients exceeding 0.9. Moreover, the predictive capacity of the multisensory system is very good, the differences between the measured and the predicted values being less than 3%. The multisensory system based on voltammetric sensors and on multivariate data analysis methods is a viable and useful tool for natural water analysis.

## 1. Introduction

The Danube is a vital river for Europe, forming one of the largest and most important water systems on the continent. The Danube has played a major role in the socioeconomic, political and cultural development of central and south-eastern Europe for centuries, and the people living in the Danube area still use this river for drinking water, irrigation, hydroelectric power, naval transport, recreation, etc. [1,2].

As a result of these activities, most of the Danube River waters had to be accumulated or regularized because agricultural fertilizers have led to an excessive growth of nutrients in the Danube and the industrial activity has significantly polluted River Danube and its tributaries [3].

Pollution is the most important problem faced by the Danube, this problem being more obvious on the Romanian territory, especially in the lower Danube sector. Pollution is caused by various factors, among which are accidental industrial waste leakage, deliberate release of pollutants, spillage of oil products from storage tanks and pipelines, use of pesticides and herbicides in agriculture, incorrect disposal of waste and household waste, solubilization of pollutants from the atmosphere, rains acid, etc. [4,5].

The concentration of water pollutants and water quality are determined by using systems based on different physico-chemical detection principles, most of these methods being standardized [6,7,8]. The systems capable of determining in situ and of reporting on-line changes in water quality need to be used in order to improve water quality monitoring, as well as to control the operations in water treatment plants [9,10]. Classical analytical methods can be used to determine water quality with high accuracy, but such methods are expensive (equipment, reagents), require long time analysis and highly qualified staff and imply off-line determinations.

Electrochemical sensors can be used for rapid screening or for in situ and on-line monitoring of several analytes concentration which are relevant for natural water pollution [11,12,13]. Simplicity, versatility and rapidity are other advantages characteristics of electrochemical methods. Most electrochemical sensors are based on potentiometry (e.g., ion selective membrane electrodes [14]) and voltamperometry (e.g., noble metals electrodes, boron doped diamond electrodes [15]). However, the determination of a limited number of parameters is not sufficient to determine water quality when analyzing complex samples such as natural waters. The use of sensor networks coupled with advanced methods for analyzing experimental data may significantly improve the results obtained [16]. These multisensory systems (also called electronic tongue) have been developed for multiple applications in various fields such as: Food [17,18,19] and pharmaceutical industry [20], as well as environmental analysis [21,22].

In a research article, Witkowska Nery et al. described a method which used paper-based potentiometric sensors (sensitive to Cl^−^, Na^+^/K^+^, Ca^2+^/Mg^2+^, NO_3_^−^) in order to discriminate water samples collected from tap or lake water and from bottled still or mineral water obtained directly from springs. A 100% correct classification of the samples was obtained by means of Principal Component Analysis (PCA) and K-nearest neighbor (KNN) methods, which prove that the system could be used for adulteration control of bottled water [23].

A potentiometric e-tongue based on lipid membranes sensors was employed to differentiate 34 water samples including still, sparkling and flavored mineral waters. The 96% correct classification rate was obtained based on the model developed by K-folds cross-validation technique. Additionally, some physico-chemical parameters, such as pH and conductivity were quantitatively estimated by using the multi-parametric model [24].

Carbó et al. describe the use of a voltammetric electronic tongue based on noble metal electrodes (iridium, rhodium, platinum, and gold) for the analysis of quality parameters in 83 spring water samples. Pulse voltammetry was the detection technique used and nitrate, sulfate, fluoride, chloride, sodium and pH were the parameters estimated. The multivariate model was based on partial least squares (PLS) analysis and showed good predictive capability for all the parameters with errors below 10% or 15% [25].

Kumar and collaborators analyzed the taste of eight packaged mineral/drinking waters, one tap water and two purified waters, obtained by reverse osmosis or deionization in commercial purification systems. The electronic tongue used was a commercial system based on potentiometric sensors. The data analysis models based on PCA, discriminant factor analysis (DFA) and soft independent modelling of class analogy (SIMCA) were able to discriminate and classify the waters according to the samples ionic content, which conditions the taste of the water [26].

Mahato and Adhikari report an electronic tongue based on functionalized polymer membrane electrodes used to monitor drinking water quality. Potentiometric sensors provide different response patterns for each drinking water sample. PCA results show good discrimination among the waters. Moreover, the monitoring of dissolved minerals was successfully achieved by using standard calibrated sensing data [27].

In one study, the experiments were conducted using an electronic tongue to monitor the drinking water quality, analyzed from the raw water in the river to the tap water of the consumer. The statistical multivariate methods of the voltammetric signals of the sensors immersed in the water samples were able to estimate the water quality [28].

Although there are numerous examples reported in various articles and studies, the global qualitative analysis of water and the quantitative determination of water parameters by using multisensor systems are still challenging, the new discoveries in this research area possibly being very important. The analytical performance of multisensor systems may be increased by using voltammetric sensors based on electroactive materials [29,30]. Such sensors may detect ionic or covalent substances, electro-active or inactive compounds, by means of various types of physical, chemical and electrochemical interactions [31,32]. The sensitivity of chemically modified sensors may be further increased by using nanomaterials such as: Carbonaceous nanomaterials (carbon nanotubes, graphene, carbon nanofibers, etc.) or nanoparticles of noble metals (Au, Pt, etc.) in the detector element [33,34]. The innovation of this work consists in the development of sensors array based on electroactive compounds and nanomaterials coupled with multivariate data analysis system, for monitoring the Danube water quality and for estimating important physico-chemical parameters from the electrochemical data. Based on our current knowledge, it is for first time when such system was used for the analysis of natural waters.

Starting from such prerequisites, the present paper focuses on a multi-sensor system based on commercial screen-printed sensors chemically modified with electroactive compounds and nanomaterials. The cyclic voltammetry was the detection method applied and the data obtained were used to construct discrimination and classification models of the water samples according to the collection point and regression models for predicting important parameters regarding the Danube water in different collection points near Galati.

## 2. Materials and Methods

### 2.1. Reagents and Solutions

KCl (Sigma-Aldrich, Saint Louis, MO, USA) and ultrapure water obtained using a Milli-Q Simplicity^®^ Water Purification System (Merk, Darmstadt, Germany) were used to prepare 0.1 M KCl solution. This was used as reference solution for checking the sensor signal before recording the signals in the water samples to be analyzed.

### 2.2. Water Samples

The water samples were taken from 7 places near Galati, as shown in Table 1.

The water samples were collected according to the current legislation in force [35]. The experimental procedure included the immersion in the mass of water of a closed container. At the depth provided in the standard procedure, the stopper was removed, the container was filled, and then it was lifted to the surface. Thereafter, the water samples for the analysis were placed in plastic vials provided with a stopper with a hermetically sealed cap. The vials for sampling were previously very well washed, rinsed with ultrapure water and dry. At the place of water sample collection, the vial was rinsed 3 times with the water to be sampled, and then filled to the refuse and the stopper was fixed so that no bubbles of air remained in the vial. The vials were transported to a laboratory where they were kept in a refrigerator until all measurements with the multi-sensor system and physico-chemical analyzes were performed.

### 2.3. Measurements with the Multisensory System

The multisensor system used in this study is based on cyclic voltammetry and was developed by the Laboratory of Sensors and Biosensors of the Lower Danube University in Galati (https://erris.gov.ro/European-Centre-of-Excellenc-1).

It consists of a potentiostat/galvanostat (Biologic SP 150, Bio-Logic Science Instruments SAS, Claix, France), a software for controlling electronic equipment and data acquisition (EC-Lab Express software V5.52, Claix, France) installed in a PC and a set of commercially screened electrodes (Metrohm-Dropsens, Llanera, Spain). The galvanostat/potentiostat applies a potential in the range between −0.4 and +0.8 V over the pseudo-reversing electrode (Ag) and records the current at the work electrode level. The current-potential-time dependence was collected for each sensor and sample to be analyzed and sent to the PC for storage and further processing.

The sensors used in this study are shown in Table 2.

The sensors used to build the system detect dissolved organic and inorganic compounds, i.e., compounds with redox properties and compounds electrochemically inactive, and ionic and covalent compounds [34,36,37]. The detection mechanism is based on the redox reactions from the sensor surface (of the electrode compounds in the solution and/or immobilized in the sensor detector element) which is accompanied by the movement of the ions in the solution to compensate for the electrical load of the sensor during potential scanning [34,36,37]. The sensors have cross-sensitivity to the compounds found in natural water, thus obtaining a chemical fingerprint of the analyzed sample.

The measurement protocol used to record the cyclic voltammograms of sensors in the Danube water samples was the following. Measurements were performed at room temperature and the scan rate was 0.1 V s^−1^. For each sensor, 5 cyclic voltammograms (CV) in 0.1 M KCl solution were recorded. The 5th CV was considered the reference CV. The sensors were removed from the KCl solution and rinsed with ultrapure water. Next, each sensor was inserted into the Danube water sample and 3 CVs were recorded, the last cycle being saved and considered the sensor response to the analyzed sample. After this, the sensor was rinsed with ultrapure water and 5 CVs were recorded again in 0.1 M KCl. The 5th CV was compared to the reference cyclic voltammogram. If the coefficient for determining the linear adjustment between the two CVs was greater than 0.9, the sensor was used for another determination. If this performance criterion has not been met, the sensor was replaced by a new sensor. The reason for choosing these CVs (the 3rd and the 5th) in the analysis is the stability and reproducibility of the sensor signal. First CVs are varying somehow because the electrochemical processes do not reach the steady state. After these first cycles, the sensors responses are highly reproducible.

All water samples were analyzed 7 times each (7 replicates), first sequentially and then randomized, with all 6 sensors and the CVs constituted the input data matrix. So, the number of files used to construct the matrix were 7 samples × 6 sensors × 7 replicates. Due to the very high number of pairs of *i,E* values from the CVs, a kernel reduction method was applied, and this method is being reported as successful in previous studies [17,38]. Briefly, the kernel method performed by means of Matlab software (v. 5.3., Mathworks, Natick, MA, USA), consist is the splitting of cyclic voltammogram in anodic and cathodic parts, the multiplication of anodic part with 10 kernel functions (bell-shaped and of unitary area), and the calculation of the areas under the anodic voltammetric curve. The 10 resulting area values, called kernel coefficients, collect the information from the cyclic voltammograms reducing the *i,E* pairs to 10 representative values for each curve. Therefore, the data resulted for the measurements with the 6 sensors consists in a matrix with 60 columns (6 sensors × 10 kernel coefficients) and 49 lines (7 water samples × 7 replicates).

The multivariate data analysis techniques used for the analysis of the experimental data were carried out using the following software: The Unscrambler (X v. 10.4, Camo, Oslo, Norway), Matlab, and Excel (v. 2010, Microsoft, Redmond, WA, USA).

### 2.4. Measurements with the Multisensory System

The physico-chemical analysis was carried out by standardized procedures (https://www.asro.ro/lista-standarde-calitatea-apei/). The pH was determined by using a pH meter (WTW, Inolab pH 7310, Weilheim, Germany), equipped with a combined glass electrode/Ag/AgCl, calibrated in three points (pH 4.01, 7.00, 10.01) (SR EN ISO 10523: 2012). Resistivity (*ρ*) and total dissolved solids (TDS) were determined with a laboratory conductometer (WTW, Inolab Cond 7310, Weilheim, Germany) (SR EN 27888: 1997). Turbidity was determined with a three-point (NFU/NTU 0.02, 10.0, and 1000) (SR EN ISO 7027-1: 2016) laboratory turbidimeter (WTW, Model TURB 430IR, Weilheim, Germany).

Determination of iron content in water with 1,10-phenanthroline was performed according to SR ISO 6332: 1996. Thus, for the determination of total iron content, hydroxylamine hydrochloride needs to be added in order to reduce iron(III) to iron(II). A solution of 1,10-phenanthroline is added to the treated water sample, and the absorbance of the red colored complex is measured at the wavelength of 510 nm.

Determination of nitrate ion in water was performed according to SR ISO 7890-3: 2000. The principle of this method consists in the spectrophotometric measurement of the absorbance of the yellow compound formed by the reaction between the sulfosalicylic acid and the nitrate in the alkaline solution.

Spectrophotometric experiments were performed with a Rayleigh UV-1601 spectrophotometer (Beijing Rayleigh Analytical Instrument Corporation, Beijing, China) equipped with a 1 cm optical length quartz cell.

### 2.5. Data Analysis

An unsupervised method, namely the main component analysis (PCA), was applied to study the data structure and to identify the sample groups. The PLS discriminant analysis (PLS-DA) was used to classify water samples according to quality or to the sampling point. The PLS1 regression method was used to estimate the correlations between the voltammetric data and the physico-chemical parameters and the predictive capacity of the sensor network.

PCA is usually the first step of data analysis. Reducing data sizes allows one to view the model by keeping as much information as possible in one’s original data. Each main component (PC) is independent (orthogonal) and it is a linear combination of variables initially measured. The first main component (PC1) accounts for the maximum of the total variance, the second (PC2) for the residual maximum variance, and so on until the total variance is taken into account. Practically, it is sufficient to keep only the components which represent a large percentage of the total variance. The correlation coefficients between the initial variables and the main components are called loadings of the main components. The values representing the samples in the space defined by the main components are called scores [39].

The Discriminant Factor Analysis (DFA) is a method based on looking for a direction along which the sample clusters are as far as possible and the samples of the same group are as close as possible. The results of the mathematical model may be represented in two or three dimensions. This method is usually used to identify and to classify unknown samples. In order to do this, an assignment function is used, which is based on the proximity of the sample to the gravity center of the group. The result of the classification is obtained by projecting the sample data on a 2D gradient of the two best discriminating functions in relation to the samples classification [40].

Detection of an electrochemical pattern for each sampling point in the Danube was done by using PLS-DA, which is a special case of partial square minimal regression for both categorical and non-quantitative variables. The 60 variables resulting from the measurements of the 49 samples with voltammetric sensors were subjected to PLS-DA regression, in which the collinearity effect of model data may be effectively reduced and the correlation between the predictive voltammetric measurements and the sample type is maximized. In order to minimize the PLS-DA regression errors, the latent variables were allocated according to the classification error rate provided by the full cross validation process [41,42].

PLS is a versatile modelling technique for multivariate data used to analyze multiple relationships between one or more sets of variables measured for a series of samples. The basic idea of PLS is to reduce the dimensionality by multiple regression by extracting latent factors which collect most of the experimental data variance, guaranteeing that the first orthogonal latent variables improve the variable prediction in the set of variables to be predicted. PLS1 is the regression method which studies a single variable explained and predicted by a set of explanatory variables [38,40].

## 3. Results and Discussion

### 3.1. Sensor Response to Water Samples

The first stage of the research was the registering of the cyclic voltammograms of all sensors in 0.1 M KCl solution, with a scan rate of 0.1 V × s^−1^. These CVs were used as reference signals for the sensor quality check (Figure S1—Supplementary Material).

In order to analyze the responses of the voltammetric sensors when exposed to water samples from the Danube, the voltammetric responses of all the sensors exposed to the same water sample and the responses of a sensor exposed to all water samples to be analyzed will be discussed.

**Figure 1:**
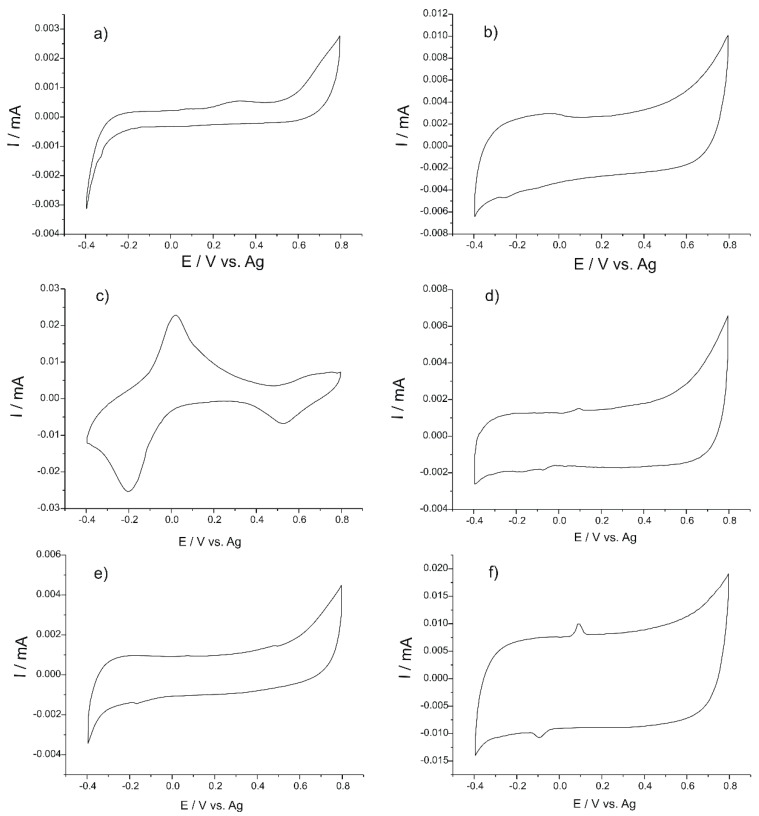
Sensor response (**a**) CoPc-SPE; (**b**) MB-SPE; (**c**) PB-SPE; (**d**) MWCNT/GNP-SPE; (**e**) MWCNF/GNP-SPE; (**f**) MWGPH/GNP-SPE exposed to the DL water sample.

Figure 1 shows the responses of all the sensors when immersed in the DL Danube water sample recorded by cyclic voltammetry in the potential range −0.4 and +0.8 V. This potential range was determined to be optimal for the achievement of stable signals of all sensors from the array.

As it may be seen in Figure 1, cyclic voltammograms show a series of peaks related to the redox processes at the surface of the sensors. These peaks are mainly due to the electroactive substances immobilized in the detector element of the sensors and to the electroactive substances present in the Danube water (e.g., cations with redox properties, phenolic compounds, etc.). On the other hand, redox processes are facilitated or hindered by the presence of other compounds in the analytical water (inhibitory or synergistic effect) and are accompanied by the movement of the ions from the solution. In addition, the presence of the nanostructured materials present in the sensing element of the sensors fosters interactions at the sensor-sample interface to analyze and transfer electrons [34,36,37,41,42,43,44].

It may be noted that each electrode produces a particular response to each of the water samples to be analyzed, which contributes to the increase of the cross-selectivity of the voltammetric sensor array.

**Figure 2:**
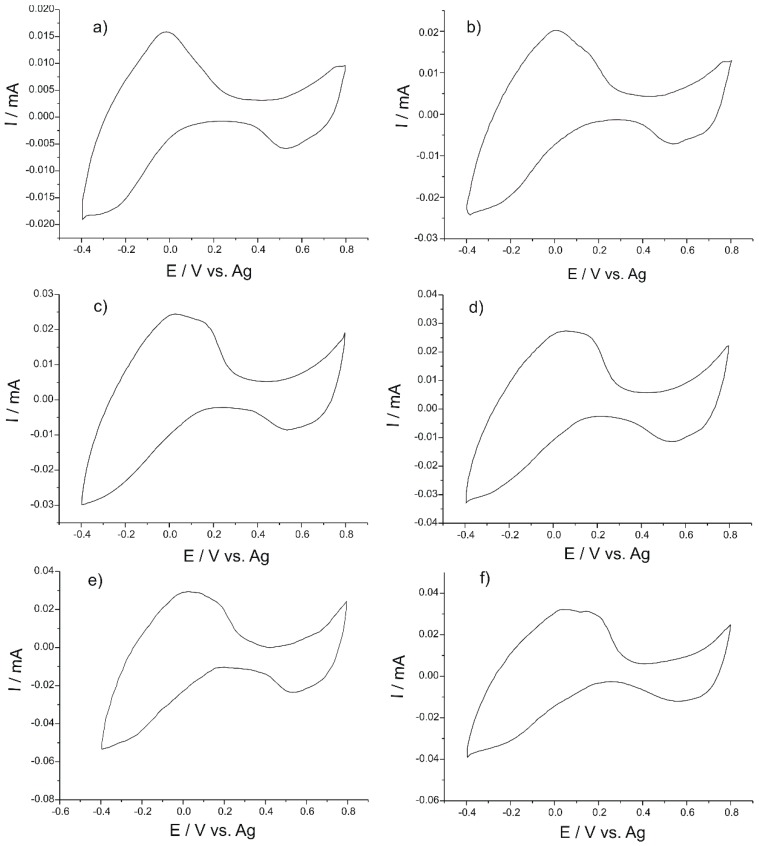
The voltammetric responses of the PB-SPE sensor exposed to all water samples (**a**) PD; (**b**) DS; (**c**) CP; (**d**) DP; (**e**) GR; (f) LN.

The cyclic voltammograms of the PB-SPE sensor exposed to all water samples are shown in Figure 2.

It may be seen that the voltammetric signals vary significantly from one sample to another. The shape of the voltamperometric curves is different, the potentials of the peaks observed are shifted to higher or lower values (±50 mV), and the peak currents have different values (±15 μA). At the most intense anodic peak in cyclic voltammograms (about 0 V), a second peak is observed in water samples (DS, DL, GR), sometimes better defined, sometimes in the form of a shoulder.

The results obtained with the other sensors immersed in the water samples are specific for each analyzed sample and sensor, constituting a chemical footprint of each water sample. It may thus be concluded that the sensor network has good cross-selectivity by providing different signals and information from the same sample to be analyzed with each system sensor. However, in order to confirm these differences, methods of multivariate data analysis should be used for water sample discrimination and classification.

### 3.2. Exploratory and Discriminant Data Analysis Models

The array of voltammetric sensors used for discriminatory studies includes the six screen-printed electrodes modified with electroactive substances (CoPc, Meldola’s Blue, Prussian Blue) and nanomaterials (MWCNT, MWCNF, MWGPH, GNP).

Since each sensor measures a specific response when immersed in each water sample, cyclic voltammograms will be used for discriminatory studies using multivariate data analysis.

In order to assess the sensitivity of the sensor network against the seven water samples coming from different points near Galati, the main components of the recorded voltammograms recorded for all the sensors towards all the water samples were analyzed.

**Figure 3:**
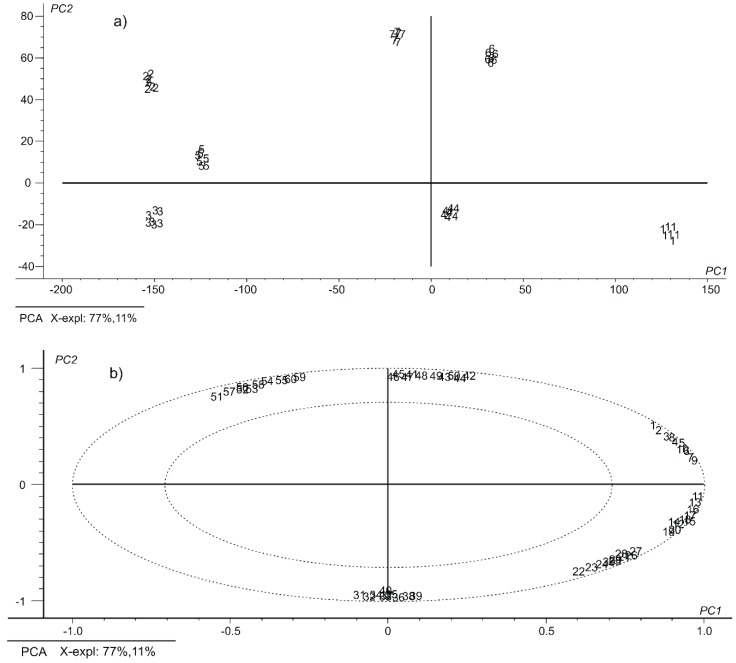
(**a**) Scores plot of the responses produced by the voltammetric sensor array when exposed to different water samples (wherein: (1) PD; (2) DS; (3) DL; (4) CP; (5) DP; (6) GR; (7) LN). (**b**) PCA loadings plot of the water samples from the voltammetric sensor array (wherein: 1–10 CoPc-SPE; 11–20 MB-SPE; 21–30 PB-SPE; 31–40 MWCNT/GNP-SPE; 41–50 MWCNF/GNP-SPE; 51–60 MWGPH/GNP-SPE).

Figure 3 presents the PCA results in the form of two-dimensional graphs of the scores and loadings.

The PCA results show a good capacity of the sensitized sensors to discriminate the Danube water collected from different sampling points near Galati. The clusters observed in the score graph (Figure 3a) correspond to the 7 replicates of the 7 water samples. It may also be seen that the total information explained by PC1 and PC2 is equal to 88%. This value is very good considering the PCA model, which explains the total variance of the model (60 components in total) in just two main components. It may be concluded that the clusters are significantly separated from one another, indicating that the seven samples of water may be discriminated with high accuracy. The spatial layout of the clusters in the score graph of the PCA is consistent with the water sampling point.

In order to determine which of the variables (i.e., of the sensors) influenced the separation of the water samples, the PCA loadings graph was analyzed. This diagram shown in Figure 3b represents the projection of the variables in the same plan as the score graph. The absolute loading value describes the contribution of the variables to the two components. The loadings graph shows that all sensors receive useful information on both main components. The sensors which caused PC1-based separation are CoPc-SPE, MB-SPE and PB-SPE, while MWCNT/GNP-SPE, MWCNF/ GNP-SPE and MWGPH/GNP contributed most to sample separation of water in PC2.

In addition, the score graph highlighted the co-relationships existing between voltammetric sensors which lead to the separation of the water samples. Considering this graph, it may be stated that there is a positive correlation between the CoPc-SPE, MB-SPE and PB-SPE sensors. On the other hand, there is a negative correlation between the MWCNT/GNP-SPE, MWCNF/GNP-SPE and MWGPH/GNP sensors.

**Figure 4:**
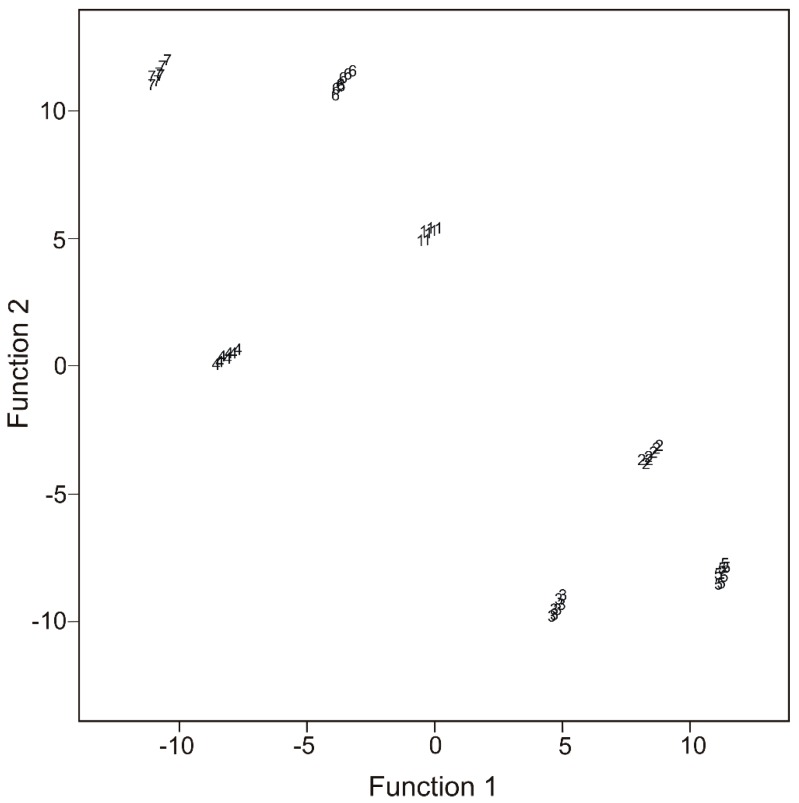
The results of the discriminant factor analysis (DFA) model.

The results of the DFA model in Figure 4 showed similar trends as in the case of PCA, the clusters corresponding to the water in different sampling points being well separated.

Within this separation, one can assume that there is a tendency to group the clusters in two macro-clusters, one corresponding to the DS, DL and DP samples (collected after the confluence with River Siret, at Libertatea Restaurant and after the confluence with River Prut), located on the left of the chart, and another group corresponding to the PD, CP, GR and LN water samples. These differences may be correlated with the impact of River Siret water, of the urban waste water and of River Prut water on the Danube water.

### 3.3. Partial Least Square-Discriminant Analysis (PLS-DA) Results

**Figure 5:**
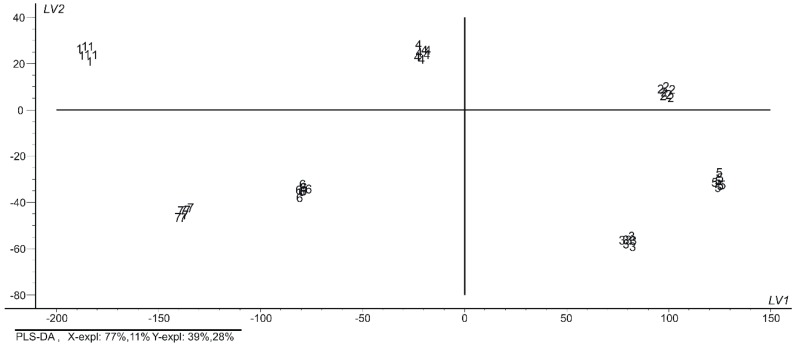
The partial least squares discriminant analysis (PLS-DA) scores plot.

In order to confirm the existence of the seven categories observed in the PCA and DFA models and the classification error in both calibration and validation, PLS-DA was performed using the total cross validation method and an optimal number of latent variants (y in this case). As it may be seen in the PLS-DA scores plot (Figure 5), the seven groups are totally separated and only two latent components explain approximately the 94% of the information.

The data on the quantification of the water samples by PLS-DA are presented in Table 3.

As shown in Table 3, both the calibration and validation values (prediction) show very good model quality (slope close to 1, offset close to 0 and a very good correlation between the sensing classification and the category variable-water quality, sampling point). In addition, very low RMSEC (root mean square error of calibration) and RMSEP (root mean square error of prediction) values are obtained indicating the predicted class membership with a value close to certainty.

The results indicate that this new methodology is able to make a very good classification of Danube waters according to their quality and to the sampling point.

### 3.4. PLS1 Regresion Results

The results of the physicochemical analyses are shown in Table 4.

Relative Standard Deviation (RSD) of the measurements were ±0.008 for the pH, ±3.2 for the TDS, ±0.01 for the *ρ*, ±1.5 for the turbidity, 0.004 for the Fe, and 0.003 for the NO_3_^−^, expressed in the same units of the physico-chemical parameters.

The quality of lower area of river Danube water, in all sampling points, is of second category of quality (“good”), in agreement with the Romanian standard of quality regarding surface waters (http://legislatie.just.ro/Public/DetaliiDocumentAfis/116349).

PLS1 regression models were designed in order to model the relationship between sensor signals (X matrix) and the physico-chemical parameters determined for the water samples (Y matrix). The X matrix is formed from 60 columns (6 sensors × 10 kernel coefficients) and 49 lines (7 samples × 7 replicates) and Y matrix for each parameter was formed by 1 column (one parameter) and 49 lines (7 samples × 7 replicates).In order to determine the relationships between the two types of experimental determinations, the sensor signals (the 10 kernel coefficients for a voltammetric curve) obtained when the sensors are immersed in the water samples are correlated with the values determined for each of the physicochemical parameters presented in Table 5.

**Figure 6:**
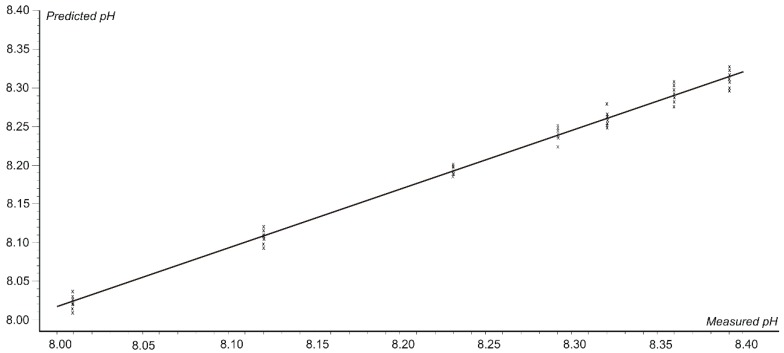
The graph of the correlation between the pH values predicted by the voltammetric sensors versus the values of the pH determined by the potentiometric method.

Figure 6 shows the graph of the correlation between the pH values predicted by the voltammetric sensors versus the parameter pH values determined by the potentiometric method.

Figure 6 shows a very good prediction based on the sensor signal values validated by the full cross validation method and the model is based on 3 latent variables. As it may be seen in Figure 6, a correlation coefficient of 0.973 with a root mean square error of correlation (RMSEC) of 1.867 at calibration and a correlation coefficient of 0.954 with an RMSEP (root mean square error of prediction) error of 2.435 are obtained.

The results obtained for the other parameters are presented in Table 5.

The correlation coefficients values higher than 0.9 obtained for all the physico-chemical parameters analyzed prove the importance of the data provided by the multisensor based voltammetric sensors in natural water analysis.

The estimated parameters from the PLS1 regression models are included in the Table 6.

The results of the physico-chemical analyses estimated from PLS1 regression models built on the data determined with voltammetric sensors (presented in Table 6) are very close to those determined experimentally, the differences being less than 3% for all the quantified parameters. Therefore, the multisensory system can be successfully used to estimate certain quantitative parameters of water samples.

## 4. Conclusions

In this study, a multisensory system was developed for monitoring the Danube water quality along sampling points and for estimating some physico-chemical parameters by using the PLS1 regression models. The parameters determined experimentally and estimated from this study (pH, TDS, resistivity, turbidity, amount of Fe and amount of NO_3_^−^) are relevant to water quality. The multisensory system used in this study is made up of carbon-based sensitized sensors modified with electroactive compounds or nanomaterials. The sensor array was built by selecting sensors with a wide variety of electrochemical responses, thus with high cross-selectivity. The detection method used was cyclic voltammetry, and PCA, DFA, and PLS-DA models based on electrochemical data showed that the multisensory system is able to discriminate and classify water samples according to the sampling point and to water quality. PLS1 regression models have shown that a series of parameters relevant to water quality may be accurately estimated from the sensor data. The method which uses the multisensory system is simple, robust, versatile, relatively inexpensive, and may be used in situ and on-line to instantly detect changes in the chemical composition of natural waters.

## Figures and Tables

**Table 1:** Water samples under study.

#	Sample	Site	Geographical Coordinates
1	PD	Before the confluence with River Siret (Priza Dunării)	45°22′ N, 28°01′ E
2	DS	After the confluence with River Siret	45°24′ N, 28°01′ E
3	DL	At Libertatea Restaurant	45°25′ N, 28°03′ E
4	CP	Before the confluence with River Prut (Cotu Pisicii)	45°25′ N, 28°11′ E
5	DP	After the confluence with River Prut	45°27′ N, 28°14′ E
6	GR	In the neighborhood of Grindu village	45°24′ N, 28°16′ E
7	LN	In the neighborhood of Luncaviţa village	45°19′ N, 28°20′ E

**Table 2:** Voltammetric sensors array.

Sensor	Abbreviation
Screen-printed Co-phthalocyanine/Carbon electrode	CoPc-SPE
Screen-printed Meldola’s Blue/Carbon electrode	MB-SPE
Screen-printed Prussian Blue/Carbon electrode	PB-SPE
Multi-Walled Carbon Nanotubes-Gold Nanoparticles modified screen-printed electrode	MWCNT/GNP-SPE
Multi-Walled Carbon Nanofibres-Gold Nanoparticles modified screen-printed electrode	MWCNF/GNP-SPE
Multi-Walled Graphene-Gold Nanoparticles modified screen-printed electrode	MWGPH/GNP-SPE

**Table 3:** Calibration and Validation PLS-DA results.

Group	Correlation Coefficient		RMSEC	RMSEP
	Calibration	Validation		
1	0.984	0.975	0.078	0.084
2	0.989	0.974	0.081	0.088
3	0.993	0.989	0.089	0.095
4	0.983	0.975	0.084	0.091
5	0.987	0.977	0.098	0.114
6	0.991	0.987	0.072	0.081
7	0.985	0.976	0.082	0.092

RMSEC (root mean square error of calibration); RMSEP (root mean square error of prediction).

**Table 4:** Results of the physico-chemical analyses.

Sample	pH	TDS/mg L^−1^	*ρ*/kΩ cm	Turbidity/NFU	Fe/mg L^−1^	NO_3_^−^/mg L^−1^
1	8.32	379	2.64	112	0.452	0.345
2	8.39	353	2.83	126	0.468	0.372
3	8.23	338	2.96	154	0.565	0.405
4	8.01	363	2.75	146	0.523	0.338
5	8.12	391	2.56	151	0.484	0.365
6	8.36	454	2.20	132	0.456	0.388
7	8.29	464	2.16	125	0.498	0.353

**Table 5:** Results of PLS1 regression models.

Parameter	Calibration		Validation	
	Correlation Coefficient	RMSEC	Correlation Coefficient	RMSEP
pH	0.973	1.867	0.954	2.435
TDS	0.987	1.758	0.966	2.125
*ρ*	0.984	1.662	0.968	1.995
Turbidity	0.974	1.889	0.955	2.235
Fe	0.992	1.744	0.970	1.744
NO_3_^−^	0.986	1.921	0.969	2.368

**Table 6:** Estimated parameters from the PLS1 regression models.

	Estimated Parameters					
Sample	pH	TDS/mg L^−1^	*ρ*/kΩ cm	Turbidity/NFU	Fe/mg L^−1^	NO_3_^−^/mg L^−1^
1	8.329	374.5	2.656	110.5	0.4572	0.3498
2	8.381	348.8	2.813	124.4	0.4626	0.3668
3	8.239	342.1	2.942	152.0	0.5715	0.3993
4	8.001	367.4	2.767	147.9	0.5290	0.3427
5	8.111	386.3	2.545	149.0	0.4896	0.3599
6	8.369	459.4	2.213	130.3	0.4508	0.3826
7	8.281	458.4	2.173	126.6	0.4923	0.3579

## Supplementary Materials

The following are available online at https://www.mdpi.com/2079-4991/9/6/891/s1, Figure S1: Sensor response (a) CoPc-SPE; (b) MB-SPE; (c) PB-SPE; (d) MWCNT/GNP-SPE; (e) MWCNF/GNP-SPE; (f) MWGPH/GNP-SPE exposed to the 0.1 M KCl aqueous solution.
