# Supplementary material for: Multisensory System Used for the Analysis of the Water in the Lower Area of River Danube
Source: Nanomaterials (Basel). 2019 Jun 17;9(6):891. doi: 10.3390/nano9060891 (PMC6630530; doi:10.3390/nano9060891)
Supplement: Supplementary file 1 [file nanomaterials-09-00891-s001.pdf]

nanomaterials

Supplementary material

Multisensory System Used for the Analysis of the Water in the Lower Area of River Danube

Constantin Apetrei *, Catalina Iticescu and Lucian Puiu Georgescu

Department of Chemistry, Physics and Environment, The European Centre of Excellence for the Environment, Faculty of Sciences and Environment, "Dunarea de Jos" University of Galati, 800008 Galati, Romania; catalina.iticescu@ugal.ro (C.I.); lucian.georgescu@ugal.ro (L.P.G.)

* Correspondence: apetreic@ugal.ro; Tel.: +40-727-580-914

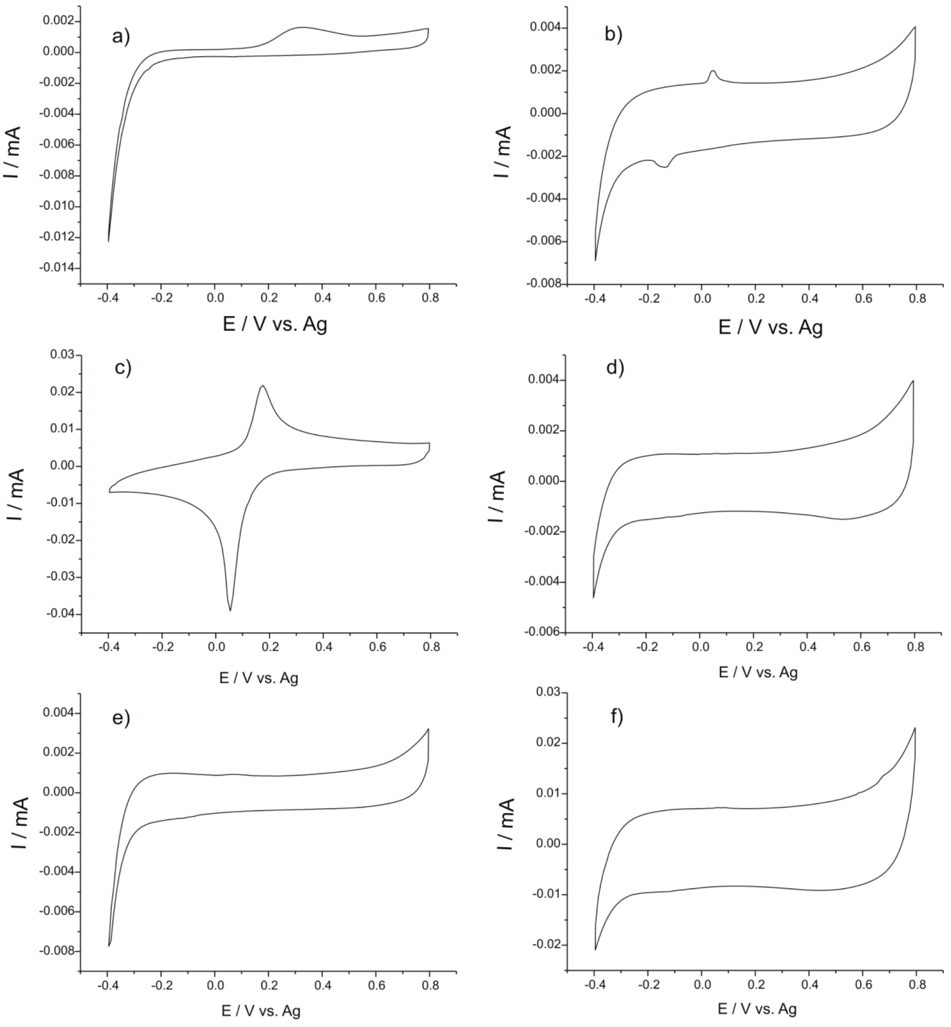

Figure S1. Sensor response **a**) CoPc-SPE; **b**) MB-SPE; **c**) PB-SPE; **d**) MWCNT/GNP-SPE; **e**) MWCNF/GNP-SPE; **f**) MWGPH/GNP-SPE exposed to the 0.1 M KCl aqueous solution.

© 2019 by the authors. Submitted for possible open access publication under the terms and conditions of the Creative Commons Attribution (CC BY) license (http://creativecommons.org/licenses/by/4.0/).
